# Special Issue: Vaccines and Vaccinations in the Pandemic

**DOI:** 10.3390/vaccines13100992

**Published:** 2025-09-23

**Authors:** Dimitrios Papagiannis, Georgios Rachiotis

**Affiliations:** 1 Public Health & Adults Immunization Lab, Department of Nursing, University of Thessaly, 41500 Larissa, Greece; 2 Department of Neurology, Medical School, University of Cyprus, Nicosia 2029, Cyprus; 3 Department of Hygiene and Epidemiology, Medical Faculty, University of Thessaly, 41222 Larissa, Greece

## 1. Background

The rapid development of multiple COVID-19 vaccines—particularly through mRNA technology, though not limited to it—within a short period following the emergence of the virus was unprecedented. This overturned long-standing theories regarding vaccine approval processes and stimulated scientific debate during and after the pandemic. However, the global effort to immunize a critical mass of the world’s population, including healthcare professionals, was challenged by the rise of misinformation, vaccine hesitancy, and the implementation of mandatory vaccination policies.

## 2. Collection of Special Issue Articles

In the Special Issue entitled “Vaccines and Vaccinations in the Pandemic Period”, 13 articles have been published (6 original papers, 1 brief report, 2 communications, 2 reviews, 1 systematic review and meta-analysis, and 1 viewpoint). For example, in the first article Pervaiz et al. reported on NSTOP officers in Pakistan, highlighting their mobilization and considerable contribution to Pakistan’s early COVID-19 response (Contribution 1).

The second article by Kim et al. discussed the global community’s preparedness for a new pandemic arising from emerging health threats. The World Health Organization (WHO) initiated the debate on the “first 100 days’ mission” in responding to a new pandemic, and several countries have already designed national contingency plans for emerging infectious threats aligned with this framework (Contribution 2). The epidemiology of many diseases shifted both during and after the pandemic. The third article by Ristić et al. presented longitudinal (1948–2023) epidemiological data on pertussis in the Autonomous Province of Vojvodina, Serbia. They found an increase in pertussis incidence rates, particularly among 10–14-year-olds, alongside a decrease in immunization coverage (Contribution 3). The fourth article in the Special Issue, by Otani et al. in their review, reported that during the COVID-19 pandemic, a decrease in the incidence of rubella occurred; however, this did not reflect a decrease in the population of susceptible individuals. On the contrary, in Japan, a reduction in the rubella vaccination rate during the COVID-19 pandemic was observed (Contribution 4). In the fifth article by Thakkar and colleagues studied the impact of vaccination campaigns on the transmission of measles in an endemic country, Somalia. Despite the considerable COVID-19-related disruptions to routine immunization services, the authors suggested that vaccination campaigns prevented a substantial burden of measles-related morbidity and mortality in Somalia (Contribution 5).

The postponement of dental care services was a major public health concern during the COVID-19 pandemic. The sixth article in the Special Issue, by Zachari et al., in a descriptive study, revealed that about one-third of dental patients postponed their dental visits, despite almost 100% of dentists being vaccinated (Contribution 6).

COVID-19 vaccine mandates constitute a controversial public health policy both in public debate and among healthcare workers (HCWs). Politis et al. from Greece at the seventh article of the Special Issue conducted a systematic review and meta-analysis of the available evidence investigating HCWs’ attitudes toward compulsory COVID-19 vaccination, both for the general population and for HCWs themselves. The findings of the study indicated that 50% of HCWs opposed COVID-19 mandatory vaccination of the general population, and 36% of them opposed vaccine mandates for HCWs (Contribution 7).

At the same time, deviations between official immunization program recommendations and the actual indications and dosages of vaccines distributed within communities are an under-investigated research topic. In eighth article of this Special Issue, Saxena et al., from the United States of America, reported deviations between the Food and Drug Administration (FDA) and the Advisory Committee on Immunization Practices (ACIP) regarding vaccine dosage recommendations. They identified five vaccines with deviations and one involving reduced dosing (Contribution 8).

Public health policies represent an important determinant of vaccination coverage. The ninth article by Juaneda et al., in a study conducted in Valencia, Spain, demonstrated a positive impact of an integrated public health policy that included public funding of vaccines and public health policy options like the co-administration of multiple core vaccines (Contribution 9). This approach demonstrated a positive impact on Meningitis B vaccination coverage (Contribution 9).

The tenth article in the Special Issue by Rasooly et al. underlined, countries that provide universal health coverage (UHC) and invest in preparedness efforts are generally better positioned to improve the overall resilience of their health systems (Contribution 10).

Vaccination coverage matters for vulnerable population groups such as individuals with substance use disorders and migrants. Mondera et al. at the eleventh article in the Special Issue presented the results of a descriptive study on COVID-19 vaccination among Italian patients suffering from substance use disorder. Notable gaps in vaccination rates were presented (20% of the participants reported no vaccination) (Contribution 11). The twelfth article by Meghani et al., from India, employed a qualitative study design to assess the effect of Partnerships in Supporting COVID-19 Vaccine Uptake. They noticed the effectiveness of strategic partnerships between health and non-health sectors in supporting vaccine uptake among migrants (Contribution 12).

Lastly, the thirteenth article in the Special Issue by Nguyen and colleagues, from the United States of America, aimed to determine whether booster vaccination influenced the frequency and severity of COVID-19 infections in the general population. In brief, booster vaccination was significantly associated with reduced risk of testing positive for COVID-19, moderate/severe illness, and Long COVID (Contribution 13).

## 3. Discussion

We sincerely hope that the present Special Issue will contribute meaningfully to the ongoing scientific discussion surrounding vaccines, vaccination strategies, and public health responses during and beyond the pandemic.

The thirteen articles included in this Special Issue collectively emphasize the multifaceted impact of COVID-19 on vaccination programs, public health policies, and disease epidemiology. The unprecedented development and deployment of COVID-19 vaccines demonstrated the potential of rapid innovation, especially with mRNA technology, but also sparked scientific and ethical debates regarding vaccine approval timelines, mandates, and public trust.

Studies from diverse settings illustrate that pandemic-related disruptions have had heterogeneous effects on routine immunization and disease incidence, with some regions experiencing significant gaps in coverage.

Vaccine hesitancy, particularly among healthcare workers, remains a significant challenge, as evidence shows substantial opposition to mandatory vaccination. This underscores the need for targeted communication strategies, public engagement, and trust-building efforts to ensure high coverage levels during health crises. Furthermore, research on policy innovations, such as integrated public health strategies and funding mechanisms, highlights the positive impacts of well-coordinated approaches in improving vaccination uptake. Importantly, vulnerable populations, including migrants and individuals with substance use disorders, continue to face barriers to immunization, necessitating equity-focused interventions and cross-sector partnerships. The findings on booster vaccination effectiveness against infection, severe disease, and Long COVID further support the continuation of booster campaigns as part of long-term pandemic management.

Taken together, the contributions in this Special Issue offer valuable insights for strengthening immunization systems, improving preparedness for future health threats, and fostering resilient, equitable public health responses.

